# Safety and Efficacy of Mechanical Thrombectomy Using Tigertriever as a Rescue Device After Failed Aspiration—Single Center Experience

**DOI:** 10.3389/fneur.2020.603679

**Published:** 2021-01-21

**Authors:** Piotr Piasecki, Marek Wierzbicki, Jerzy Narloch

**Affiliations:** Interventional Radiology Department, Military Institute of Medicine, Warsaw, Poland

**Keywords:** stroke, aspiration, thrombectomy, Tigertriver, rescue treatment

## Abstract

**Introduction:** We evaluated the safety and efficacy of a new stent retriever—Tigertriever—after failed aspiration.

**Materials and Methods:** Patients with acute ischemic stroke treated with Tigertriever between January 2018 and March 2020 were included in the study. Treatment results of Tigertriever in rescue therapy (after failed aspiration) were evaluated. Periprocedural data were retrospectively analyzed.

**Results:** Thirty patients were treated with Tigertriever (14M/16F). There were 20 rescue thrombectomies after failed aspiration. Tigertriver successful recanalization rate (mTICI ≥ 2B) was 70%: 65% in rescue therapy and 80% in first-line therapy. The type of first line treatment had no impact on mRS after 1 month and 3 months (ns). There was significant improvement in NIHSS in all patients (mean NIHSS: 17 vs. 10, *p* = 0.028), in rescue treatment (mean NIHSS: 17 vs. 11, *p* = 0.048) and in first line treatment (mean NIHSS: 16 vs. 8, *p* = 0.0005). Better results in NIHSS at discharge were linked with first pass success (*p* = 0.002), better mTICI at the end of the procedure (*p* = 0.0006), and administration of rtPA (*p* = 0.013).

**Conclusions:** The new stent retriever Tigertriever is an efficient and safe tool to be used as a rescue device after an unsuccessful first line aspiration technique.

## Introduction

**Figure 1:**
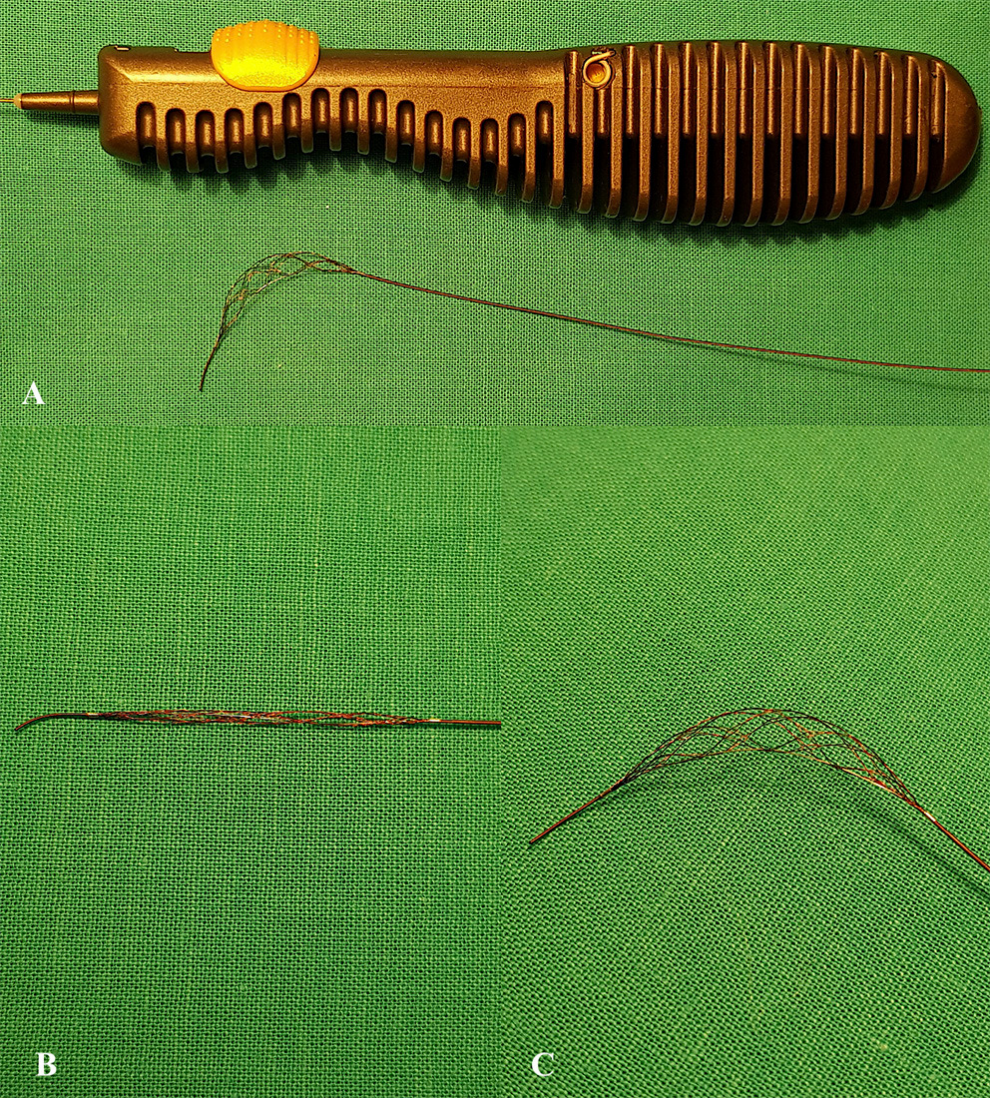
**(A)** Tigertriever device with open mesh structure using integrated handle slider. Magnified images of the Tigertriever mesh in closed **(B)** and open **(C)** positions.

**Figure 2:**
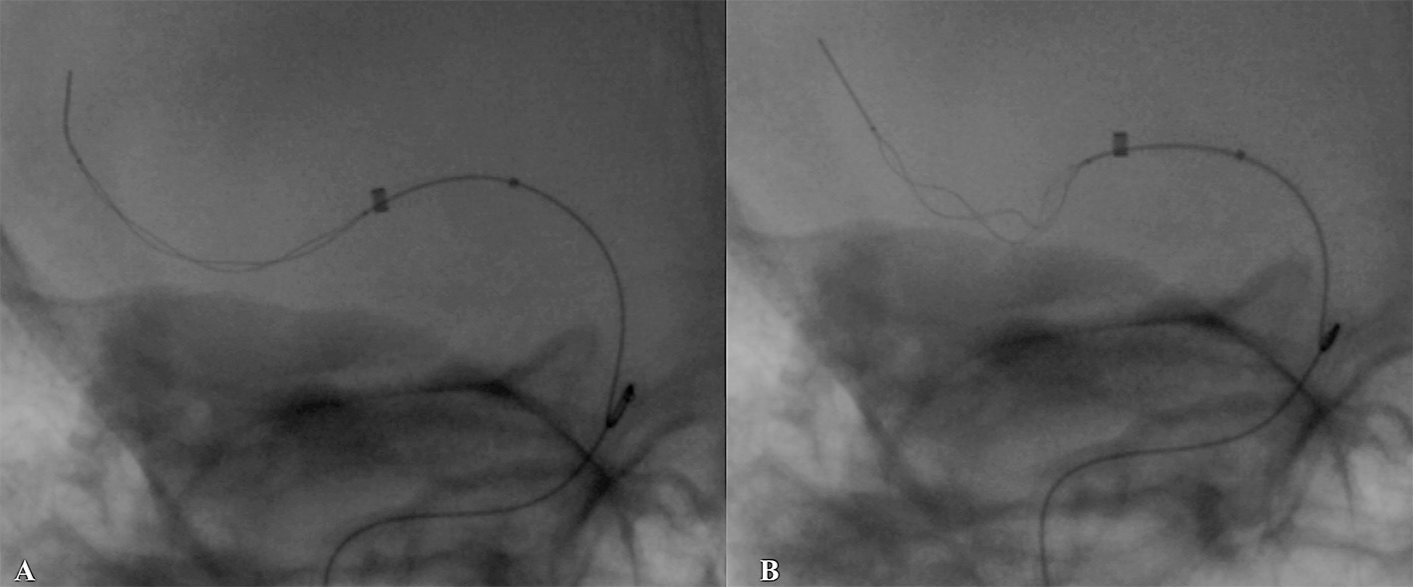
Native (unsubtracted) images of Tigertriever behavior during retrieval attempt. **(A)** Stent deployment at occlusion site, **(B)** improved expansion using repetitive inflation–deflation technique, stent retriever position during retraction with a bow-tie configuration of the stent mesh, which gives a highly improved vessel wall apposition.

In recent years, endovascular treatment for large vessel occlusion in stroke patients has become more effective and safer (1, 2). Currently, the first line techniques to remove embolic material from cerebral arteries are thrombectomy with stent retrievers (mechanical thrombectomy—MT) and aspiration (direct aspiration fist pass technique—ADAPT) (2–4). The selection of one of these techniques depends on the discretion of an interventionalist. In the ADAPT technique, the clot is removed by a large lumen distal catheter using a dedicated vacuum pump, which provides continuous suction during the procedure (2). The ASTER trial showed no significant differences in successful revascularization and results after 3 months of follow-up between an aspiration technique alone and a first-line stent retriever treatment (3–5). Rapid technological developments have led to the substitution of older-generation MT devices by new equipment which use a novel construction of the stent structure (6). One of the newest devices is the Tigertriever (Rapid Medical, Yoqneam, Israel) (7, 8), of which there is scarce clinical data on its safety and efficacy (7, 9). Tigertriever “is a stent retriever comprising a collapsible, non-detachable, fully retrievable, wire braided construction attached to a 180 cm pusher wire. The stent construction is expanded by pulling a core wire, which is connected to the distal end of the mesh. The proximal end of the core wire is connected to a slider in the handle. The operator can expand and contract the mesh to conform properly to the diameter of the affected vessel wall. Because the wires of the mesh are completely radiopaque, the device can be seen in its entirety under fluoroscopy” (7). These properties may enable better control of stent opening, resulting in an improved apposition to the vessel wall with adjustable radial force, that in effect, may improve the results of mechanical thrombectomy (Figures 1, 2). There are 3 versions of the Tigertriever. First, the standard Tigertriever is delivered through a 0.021″ microcatheter and is usually used in larger arteries [i.e., the internal carotid artery (ICA)]. The second type, Tigertriever 17, is delivered through a 0.017″ microcatheter and is used mainly for occlusions of the middle cerebral and anterior cerebral arteries. Finally, Tigertriever 13, the smallest version, is designed for reaching distal occlusion sites (7, 9). In some cases, the complexity of a thrombus can render aspiration inefficient. In this situation, a stent retriever is required. To date, there is no data from any randomized trial that supports a specific device to be used in a rescue treatment (1). It can be assumed that in such cases, the thrombectomy may be more difficult due to higher clot integrity, vessel tortuosity, or other factors. For this purpose, we could use stent retrievers that have a well-established efficiency in first line treatment or aim to identify a novel device with promising features that may lead to successful rescue thrombectomies (1).

We decided to select the novel device the Tigertriever, due to its unique construction, securing a direct impact of the operator on stent mesh size during the procedure. It is the only device with such properties within a wide range of stent retrievers (7, 8). The aim of this study was to evaluate the safety and efficacy of Tigertriever as a rescue device after failed aspiration. Our research may be valuable to many centers using the ADAPT technique in stroke treatment and aiming to identify an optimal treatment option in case of a failed aspiration. To the best of our knowledge this topic is yet to be assessed.

## Materials and Methods

### Study Design

Patients with acute ischemic stroke treated with Tigertriever between January 2019 and March 2020 were included in the study. Tigertriever-treated patients comprised a group of consecutive cases in the comprehensive stroke center (150 cases/year). Treatment results of Tigertriever in rescue therapy (after failed aspiration) and in first-line therapy were evaluated. Periprocedural data and clinical outcomes of the two groups of patients were retrospectively analyzed. The following data were evaluated: time from onset-to-groin, time from onset-to-recanalization, time of the procedure, recanalization rate, first pass success rate, periprocedural complications rate, National Institutes of Health Stroke Scale score (NIHSS) value before and after treatment, and modified Rankin Scale (mRS) score at 1 and 3 months after the procedure.

### Ethics Approval Statement

This study was reviewed and approved by the authors' institutional review board (decision number 34/WIM/2020) and procedures followed were in accordance with institutional guidelines and with the 1964 Helsinki Declaration and its later amendments or comparable ethical standards.

### Patient Selection and Eligibility Criteria

Patients with acute ischemic stroke qualified for endovascular treatment after assessment with non-enhanced computed tomography (NECT) and intracranial vessels computed tomography angiography (CTA) according to the AHA/ASA (American Heart Association/American Stroke Association) and ESO-ESMINT (European Stroke Organization—European Society of Minimally Invasive Neurological Therapy) guidelines (10, 11). Based on the ESO/AHA guidelines, patients received intravenous recombinant tissue plasminogen activator (rtPA, 0.9 mg/kg) (10, 12). A mechanical thrombectomy (MT) was performed under local or general anesthesia [at the discretion of the anesthesiologist, depending on patient cooperation and Glasgow Coma Scale (GCS) status].

When appropriate, the rescue use of Tigertriever, after 3 unsuccessful attempts of aspiration, was performed.

To minimize MT complications, the procedure was limited to 3 attempts of Tigertriever use. The effects of MT were evaluated according to the modified Thrombolysis in Cerebral Infarction (mTICI) score and 2B/2C/3 scores were considered successful (13). Neurological status was evaluated with NIHSS estimated at the time of MT qualification on admission and on the day of discharge. The mRS score was evaluated at 1 month and 3 months after the procedure. We recorded the times from onset to groin, from admission to our stroke center to the needle insertion (door-to-groin), from the onset of stroke to recanalization of the target vessel (onset-to-recanalization). If possible, dependent on the patient's clinical condition, written consent was taken. To evaluate any early complications of MT, head NECT was performed 24 h after MT or earlier when neurological status deteriorated (14). All radiological examinations were assessed by trained radiologists (the authors) by consensus.

### Endovascular Procedure/Intervention

First pass treatment (ADAPT or Tigertriever) was chosen by the interventional radiologist. In case of unsuccessful attempts of aspiration (maximum 3 attempts with ADAPT), Tigertriever was used for rescue thrombectomy. To minimize MT complications, the procedure was limited to 3 attempts of Tigertriever passes. No other device was used.

### Tigertriever Thrombectomy—a Technical Note

The procedure was preceded by angiographic examination using an 8F Radifocus Introducer Sheath (Terumo, Tokyo, Japan) and an Impress diagnostic catheter (Merit Medical, South Jordan, Utah, USA) to confirm cerebral large vessel occlusion (LVO). Then, a Neuron MAX (Penumbra, Inc., Alameda, California, USA) guiding catheter was used. An intermediate catheter (Navien 0.072″, Medtronic, Minnesota, USA or Catalyst 6, Stryker, Fremont, California, USA) and a 0.017″ or 0.021″ microcatheter (Headway, Microvention, California, USA) with a 0.014″ microwire (Traxcess, Terumo, Tokyo, Japan) were placed close to the thrombus. After verifying the microcatheter position beyond the thrombus by a short injection of contrast medium, the Tigertriever was deployed at the occlusion using the “push and pull” technique. In order to anchor the Tigertriever to the thrombus, two techniques could be used. First, the “conservative” technique, based on expanding the stent for 5 min before retrieval. Or second, the “massage” technique—repetitive inflation-deflation of the stent retriever with a stepped ratchet handle (7). Withdrawal of the stent would ensue after the optimal expansion of the stent against the vessel wall and with continuous pump-facilitated aspiration.

First pass success recanalization was considered as a result if mTICI ≥ 2B after a single Tigertriver pass. The protocol of the procedure adopted in the department did not allow for more than 3 passes with a stent-retriever, in order to minimize hemorrhagic complications. No other stent-retrievers were used.

### ADAPT Procedure

The first-pass ADAPT technique was performed using the same diagnostic and distal access technique with a Neuron MAX (Penumbra, Inc., Alameda, California, USA) guiding catheter. Subsequently, contact aspiration catheters (ACE68, Penumbra, Inc., Alameda, California, USA) were advanced into direct contact with the thrombus. The Penumbra Aspiration system was used with a constant automatic aspiration from Pump MAX (Penumbra, Inc., Alameda, California, USA) (15). After 3 failed attempts of aspiration, Tigertriever was chosen as a rescue thrombectomy option.

### Statistical Analysis

Recanalization rates, number of attempts, first-pass rates, and times of the procedure were calculated for both groups. To make statistical comparisons of the two groups (first line Tigertriever vs. failed aspiration + Tigertriever) we used the Wilcoxon signed-rank test and Mann-Whitney *U* test. The Fisher exact test was used for some categorical data. *P* values < 0.05 were considered to be statistically significant. Multivariate analysis for first pass reperfusion with Tigertriever use was not executed due to methodological reasons—small sample size in the group would render imprecise predictions, thus analysis was not pursued. For some nominal categorical data, multiple correspondence analysis (MCA) was carried out. MCA graphically represents the association between two or more qualitative variables. The results are interpreted on the basis of the relative positions of the points and their distribution along the dimensions; as categories become more similar in distribution, the closer they are represented in space.

Receiver operating characteristic (ROC) curves were analyzed for the NIHSS. Statistical calculations were carried out using the STATISTICA 12.0 software (TIBCO Software, Palo Alto, California).

## Results

### Overall Patients' Results

We retrospectively evaluated the records of 30 patients treated in our center with Tigertriever (14 males and 16 females) due to acute ischemic stroke. There were 20 (67%) rescue Tigertriever thrombectomies after failed aspiration. Patients' detailed characteristics and clinical data are presented in Table 1. Eighteen patients (60%) received intravenous rtPA before MT. The overall successful recanalization rate (mTICI ≥ 2B) was 70% in 13 (65%) patients in rescue therapy and 8 (80%) patients treated with Tigertriever as first line therapy. The median time from groin puncture to successful recanalization was 57 min. There was no significant difference in the time from onset to recanalization between the groups (Tigertriver: first line vs. rescue therapy; *p* = 0.447). A shorter time from groin to recanalization resulted in a better outcome (mRS ≤ 2) after 3 months (*p* = 0.015, R = −0.55). The successful recanalization rate (mTICI ≥ 2B) and 3-month mortality were not dependent on the duration of the MT procedure (*p* = 0.562 and *p* = 0.280, respectively).

**Table 1:** Patient characteristics.

**Characteristics**	**All patients**	**TIGERTRIEVER rescue therapy after failed ADAPT**	**First-Line TIGERTRIEVER**	***p-value***
	30 (100%)	20/30 (67%)	10/30 (33%)	
**Baseline demographics and medical history**				
Age, mean, (years)	66	64	71	0.230^a^
Men, No. /total (%)	14/30 (47)	9/20 (45)	5/10 (50)	0.840^a^
Female No. /total (%)	16/30 (53)	11/20 (55)	5/10 (50)	0.840^a^
**Medical history, No. /total (%)**				
Hypertension	19/30 (63)	11/20 (55)	8/10 (80)	nd
Diabetes	5/30 (17)	3/20 (15)	2/10 (20)	nd
Hypercholesterolemia	11/30 (37)	6/20 (30)	5/10 (50)	nd
Current smoking	7/30 (23)	3/20 (15)	4/10 (40)	nd
Coronary artery disease	9/30 (30)	7/20 (35)	2/10 (20)	nd
Previous stroke or transient	2/30 (7)	2/20 (10)	0	nd
Previous antithrombotic medications:				
Antiplatelets	8/30 (27)	4/20 (20)	4/10 (40)	0.397^a^
Anticoagulants	5/30 (17)	3/20 (15)	2/10 (20)	0.845^a^
**Current stroke event**				
National Institutes of Health Stroke Scale (NIHSS) score on admission, mean (SD)	17 (5.53)	17 (5.54)	16 (5.68)	0.226^a^
Pre-stroke modified Rankin Scale score, No. /total (%):				
0	14/30 (47)	10/20 (50)	4/10 (40)	0.745^a^
1	13/30 (43)	8/20 (40)	5/10 (50)	0.745^a^
2	1/30 (3)	1/20 (5)	0	0.745^a^
3	0	0	0	nd
>3	2/30 (7 )	1/20 (5 )	1/10 (10 )	0.745^a^
Intravenous recombinant tissue plasminogen activator, No. /total (%)	18/30 (60)	13/20 (65)	5/10 (50)	1.0^b^
General anesthesia, No. /total (%)	11/30 (37)	9/20 (45)	2/10 (20)	0.246^b^
**Site of occlusion, No. /total (%)**				
Middle cerebral artery branch M1	10/30 (33)	7/20 (35)	3/10 (30)	0.913^a^
Middle cerebral artery branch M2	10/30 (33)	5/20 (23)	5/10 (50)	0.913^a^
Intracranial internal carotid artery	3/30 (10)	3/20 (15)	0	0.913^a^
Tandem lesion	5/30 (17)	3/20 (15)	2/10 (20)	0.913^a^
Basilar artery	2/30 (7)	2/20 (10)	0	0.913^a^
**Median time frames (min)**				
Onset to groin puncture time, median, min	271	276	268	0.649^a^
Door (angio-suite) to groin puncture, median, min	31	32	31	0.373^a^
Onset to revascularization, median, min	334	333	336	0.447^a^
Groin to revascularization, median, min	57	57	56	1.0^a^
**Outcomes**				
Number of passes with Tigertriever No. /total (%)	39 (100)	28/39 (72)	11/39 (28)	nd
Successful revascularization at the end of all procedures mTICI score of 2B/2C/3, No. /total (%)	21/30 (70)	13/20 (65)	8/10 (80)	0.812^a^
Successful first pass Tigertriver revascularization mTICI 2B/2C/3, No. /total (%)	16/30 (53)	11/20 (55)	5/10 (50)	1.0^c^
**Clinical Efficacy Outcomes**				
NIHSS score at discharge, mean	10	11	8	0.197^a^
Functional independence at 1 month (mRS score of 2 or lower), No. /total (%)	8/30 (27)	5/20 (25)	3/10 (30)	1.0^b^
Functional independence at 3 months (mRS score of 2 or lower), No. /total (%)	9/30 (30)	6/20 (30)	3/10 (30)	1.0^b^
**Adverse Events**				
All-cause mortality at 1 month, No. /total (%)	7/30 (23)	6/20 (30,0)	1/10 (10)	0.371^b^
All-cause mortality at 3 months, No. /total (%)	9/30 (30)	8/20 (40)	1/10 (10)	0.203^b^
Intracranial hemorrhage at 24 h after MT, No. /total (%):	15/30 (50)	11/20 (55)	4/10 (40)	0.548^b^
Hemorrhagic infarction Type 1	10/30 (33)	8/20 (40)	2/10 (20)	nd
Hemorrhagic infarction Type 2	5/30 (20)	3/20 (20)	2/10 (20)	nd
Parenchymal hematoma	0	0	0	
Intraventricular hemorrhage	0	0	0	
Subarachnoid hemorrhage	1/30 (3)	1/20 (5)	0	nd

a *Mann-Whitney U test*.

b *Wilcoxon signed-rank test*.

c *Fischer exact test*.

**Figure 3:**
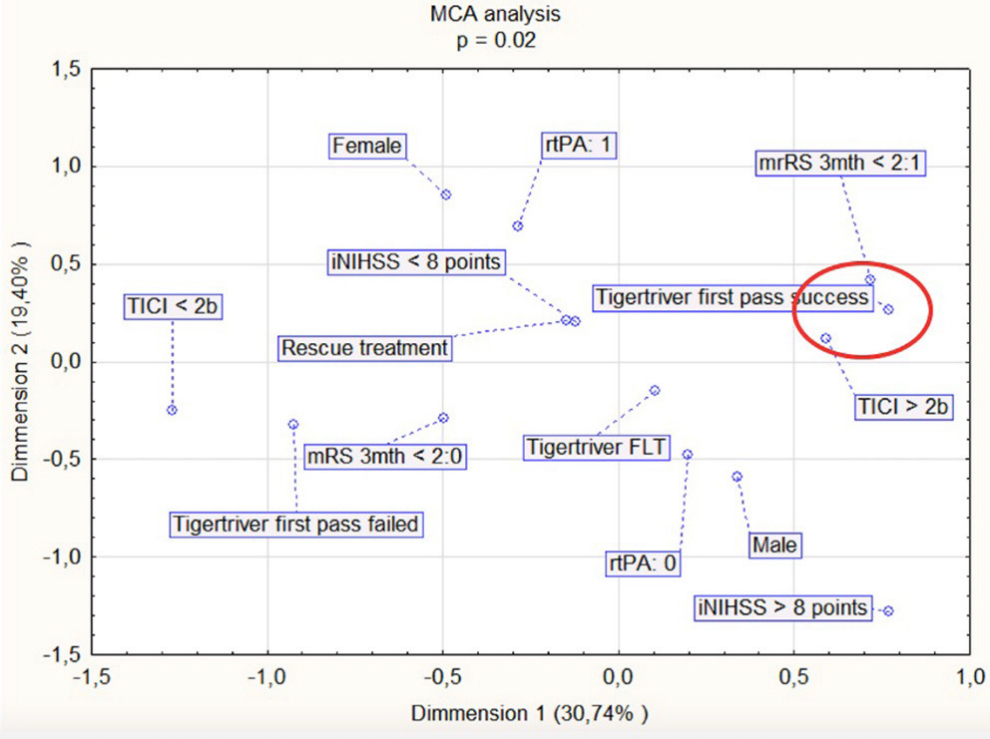
Multiple correspondence analysis (MCA) for factors associated with an improved mRS (modified Rankin Scale) at 3 months (encircled in red). iNIHSS, NIHSS at admission; Tigertriever FLT, first line Tigertriever treatment.

There was no difference in the initial NIHSS between patients in the Tigertriever rescue treatment group and first line treatment group (17 vs. 16, respectively, *p* = 0.226). There was significant improvement in NIHSS in all patients (mean NIHSS before and after treatment: 17 vs. 10, *p* = 0.028), in rescue treatment (mean NIHSS: 17 vs. 11, *p* = 0.048), and in first line treatment (mean NIHSS: 16 vs. 8, *p* = 0.0005). A lower NIHSS at discharge was linked with Tigertriever first pass success (*p* = 0.002), better mTICI at the end of the procedure (*p* = 0.0006), administration of rtPA (*p* = 0.013), and treatment under local anesthesia (*p* = 0.003). Nine patients (30%) presented good clinical outcomes (mRS score 0–2) at 3 months follow up. An improved mRS at 3 months was observed in patients with Tigertriever first pass success and TICI ≥ 2B according to MCA analysis (*p* = 0.0001) (Figure 3). The type of first line treatment had no impact on mRS after 1 month and 3 months (*p* = 0.806 and *p* = 0.859, respectively). NIHSS status did not determine the type of anesthesia (*p* = 0.444). There were no significant differences in clinical outcomes (as evaluated by mRS scale) between the 19 patients (63%) treated under local anesthesia and the 11 patients (37%) under general anesthesia.

### Adverse Events and Death Rates

There was a single case of subarachnoid hemorrhage in the ADAPT group in a 63-year-old female, who died due to the complication of acquired massive pneumonia. There were 15 cases of intracranial hemorrhagic transformation revealed by CT 24 h after thrombectomy (Table 1). There were no emboli in new vascular territory.

There were 7 deaths after 1 month and 9 after 3 months. Three-month mortality was correlated with NIHSS before treatment (*p* = 0.035, R = 0.75) with a cut-off of 8 points based on ROC (*p* = 0.025). Patients with an NIHSS higher than 8 were more likely to die at 3 months post procedure (OR-0.66, 95% CI- 0.05-8.16, *p* = 0.75). None of these cases were related directly to the endovascular procedure. There was no significant difference in the 1-month and 3- month death rate between patient groups treated with Tigertriever as first line therapy and as a rescue device (*p* = 0.371 and *p* = 0.203, respectively).

### Tigertriever as a Rescue Device After Unsuccessful ADAPT

Twenty patients (9 male and 11 female) were treated with Tigertriever after unsuccessful ADAPT. The age of patients was between 39 and 81 years (mean 64 years). The mean NIHSS value on admission was 17. Intravenous rtPA before MT was administered to 13 patients (65%). Nine patients (45%) were treated under general anesthesia. There were 28 Tigertriever passes in 20 patients (1 pass in 13 patients, 2 passes in 6 patients and 3 passes in 1 patient). Successful recanalization (mTICI ≥ 2B) at the end of the procedure was achieved in 13 patients (65%), including 11 first-pass recanalizations. The mean NIHSS score at discharge was 11. Six patients (30%) achieved a good clinical outcome (mRS score ≤ 2) after 3 months.

### First-line Stent Retriever Tigertriever (SRFL) Results

Ten patients (5 male and 5 female) were treated with Tigertriever as a first line device for MT. The age of patients in this group was between 27 and 88 years (mean 70 years). The mean NIHSS on admission was 16. Intravenous rtPA before MT was administered in 5 patients. Two patients (20%) were treated under general anesthesia. There were 11 Tigertriver passes in 10 patients (1 pass in 9 patients, 2 passes in 1 patient). Successful recanalization (mTICI ≥ 2B) at the end of the procedure was achieved in 8 patients (80%) and in 5 patients (50%) including first-pass Tigertriver recanalizations. The mean NIHSS score at discharge was 8. Three patients (30%) presented with good clinical outcome (mRS score 0-2) after 3 months.

## Discussion

The ADAPT technique has proved to be effective in achieving angiographic results of mTICI ≥ 2B in the majority of large vessel occlusion cases (5). In the event of repeated failed aspiration, a salvage technique is required. The use of stent-retriever devices in MT is the obvious choice in these cases. There is a wide portfolio of devices for MT, with few having been scientifically proven to be effective (1, 16–18).

We choose the Tigertriever device for its unique characteristics. Firstly, its mechanism enables the interventional neuroradiologist to actively adjust the size of the mesh to the vessel and its tortuosity during the procedure. Secondly, it is possible to achieve better thrombus integration with Tigertriever mesh using the “massage technique” (7). This feature may be crucial in achieving successful recanalization after failed aspiration attempts, which could be linked to thrombus composition. Another reason for the use of Tigertriever was a high rate of successful recanalization in first line treatment, reported to be 75.4% by Kara et al. (7)

Within our study population, the Tigertriever success rate was 70% (80% in first line treatment and 65% in rescue thrombectomy). First attempt success rate and safety profile were also satisfactory. The use of Tigertriever as a rescue option after 3 aspiration attempts gave us comparable results to other reports on first line treatment. This is an important observation as a substantial amount of time is required to switch from ADAPT to stent retriever thrombectomy, which is also affected by a more complex clot structure. Earlier research has raised a concern that efficient stent retriever thrombectomy may be delayed in cases of failed aspiration, advocating the use of stent-retriever alone. Other researchers have reported a shorter procedure time when using ADAPT compared to stent retriever thrombectomy (5, 19). Here, we did not find any significant differences in time from groin puncture to recanalization between first line and rescue Tigertriever groups (56 min vs. 57 min, *p* > 0.05). The relatively shorter time of ADAPT alone, compared to stent retriever thrombectomy, compensates for the need of the conversion to stent retriever. Neither of the results of MT were affected by the first line use of ADAPT. Previous data, mainly based on single center experiences, has given mixed results regarding aspiration success rates, the impact on patient's neurological status, and rates of embolization in new territory (20, 21). A systemic review of stroke treatment outcomes in over 9,000 patients by Primiani et al. showed there were no significant differences in successful recanalization rates and mRS at 90 days post procedure between ADAPT and stent retriever thrombectomy (22). In the randomized multicenter ASTER trial, Lapergue et al. did not report significant differences in successful treatment rates or mRS after 3 months (4). These data are supported by the meta-analysis by Qin et al., who highlighted an improved mRS after 90 days in the aspiration group (23). In our study, NIHSS was significantly improved after the procedure for the investigated group overall (median NIHSS 17 vs. 10, *p* = 0.028) and was better after the first pass success in patients who also received rtPA.

There were no significant differences in mRS score and mortality rate at 3 months follow-up between Tigertriever used as first choice and as a rescue treatment. Nine patients had died at 3 months follow-up, 8 in the aspiration group and 1 in Tigertriever used in first-line treatment group (*p* > 0.05).

Ducroux et al. did not find any differences in the first pass success rate between aspiration and stent retrievers (31 vs. 26%, *p* = 0.44) (19). First pass success was also associated with significantly better clinical outcomes and lower mortality. Furthermore, it also led to a lower rate of periprocedural adverse events (19). In this study, we report a 53% first pass success rate, 11/20 (55%) when Tigertriever was used as a rescue therapy and 5/10 (50%) when used in first line thrombectomy. First pass success with Tigertriever led to better thrombectomy results and improved mRS at 3 months. There was no device-related impact on mortality at 3 months follow-up.

Kara et al. reported a successful first pass Tigertriever attempt in 38% patients. The main goal of their study was to compare 2 different approaches of Tigertriever thrombectomy, a standard unsheathing technique (with 32% first pass success) and repeated inflation-deflation (“massage”) technique (with 48% first pass success) (7). Our observations support the fact that the ability to change stent mesh size during thrombectomy could be advantageous in rescue thrombectomy, when a more complex and difficult clot is expected. Yet, there have been no head-to-head comparisons of Tigertriever with other stent retrievers to date.

Failure to retrieve the thrombus could be attributed to: (a) inability to cross the thrombus with the Tigertriever microcatheter or (b) the inability to remove the thrombus from the cerebral artery. In our study group, the first possibility did not occur. All 9 failures (2 in first-line treatment and 7 in salvage treatment) were failed attempts to remove the thrombus. The most likely cause was its composition—i.e., hard cardiovascular thrombus. This is indirectly supported by the fact that in 3 cases the operator noticed incomplete opening of the Tigertriever at the site of the thrombus—there was modeling of the Tigertriever mesh resulting in incomplete vessel wall apposition. The stenosis caused by the thrombus produced excessive tension of the system while pulling, which could damage the vessel wall. Therefore, in these cases the size of the Tigertriever was deflated in order to safely remove the device. In the remaining failed cases, no or fragmented thrombi were found attached to the Tigertriever mesh.

A single case of subarachnoid hemorrhage was noted in 30 procedures. It concerned a 63-years old female, with internal carotid T-occlusion, who received the full dose of rtPA. After 3 unsuccessful aspirations, mTICI 3 was restored using single pass of Tigertriver 17. There were no significant technical difficulties (e.g., increased resistance) or direct features of contrast extravasation. However, the control CT examination revealed SAH, HI-2 in the middle cerebral artery vascular region. The thrombus involved the M2/M3 segment of the middle cerebral artery, the most probable cause of SAH was damage to small perforators located in this area during traction. HI-2 hemorrhagic transformation could be attributed to damage to the blood-brain barrier (onset-to reperfusion −6 h 37 min). The patient died on the 6th day due to the complication of massive pneumonia.

The retrospective design and relatively small number of patients are the main limitations of this study. However, the amount of clinical and treatment data fulfilled the criteria to perform statistical analysis. Choosing Tigertriever as first line therapy was done at the discretion of the interventional radiologist.

In conclusion, the new stent retriever Tigertriever is an efficient and safe tool to be used as a rescue device after an unsuccessful first line aspiration technique. Further investigation with a larger population is required to fully determine its place in rescue thrombectomy.

## Data Availability Statement

The original contributions generated for the study are included in the article/ further inquiries can be directed to the corresponding author/s.

## Ethics Statement

The studies involving human participants were reviewed and approved by Ethical Board of Military Institute of Medicine, Warsaw, PL. Written informed consent for participation was not required for this study in accordance with the national legislation and the institutional requirements.

## Author Contributions

PP study conception and design, data analysis, statistical analysis, writing–original draft and review, study supervision. MW data collections and analysis, writing–original draft, images and tables preparation. JN data collections and analysis, writing–review. All authors read and approved final version of this manuscript.

## Conflict of Interest

The authors declare that the research was conducted in the absence of any commercial or financial relationships that could be construed as a potential conflict of interest.

## References

[1] Balami JS Sutherland BA Edmunds LD Grunwald IQ Neuhaus AA Hadley G. A systematic review and meta-analysis of randomized controlled trials of endovascular thrombectomy compared with best medical treatment for acute ischemic stroke. Int J Stroke. (2015) 10:1168–78. 10.1111/ijs.12618 26310289 PMC5102634

[2] Flynn D Francis R Halvorsrud K Gonzalo-Almorox E Craig D Robalino S. Intra-arterial mechanical thrombectomy stent retrievers and aspiration devices in the treatment of acute ischaemic stroke: a systematic review and meta-analysis with trial sequential analysis. Eur Stroke J. (2017) 2:308–18. 10.1177/2396987317719362 31008323 PMC6453187

[3] Gory B Lapergue B Blanc R Labreuche J Ben Machaa M Duhamel A. Contact aspiration versus stent retriever in patients with acute ischemic stroke with M2 occlusion in the ASTER randomized trial (Contact aspiration versus stent retriever for successful revascularization). Stroke. (2018) 49:461–4. 10.1161/STROKEAHA.117.019598 29284735

[4] Lapergue B Blanc R Gory B Labreuche J Duhamel A Marnat G. Effect of endovascular contact aspiration vs stent retriever on revascularization in patients with acute ischemic stroke and large vessel occlusion: the ASTER randomized clinical trial. JAMA. (2017) 318:443–52. 10.1001/jama.2017.9644 28763550 PMC5817613

[5] Lapergue B Labreuche J Blanc R Barreau X Berge J Consoli A. First-line use of contact aspiration for thrombectomy versus a stent retriever for recanalization in acute cerebral infarction: the randomized ASTER study protocol. Int J Stroke. (2018) 13:87–95. 10.1177/1747493017711948 28592218

[6] Nogueira RG Zaidat OO Castonguay AC Haussen DC Martin CO Holloway WE. Rescue thrombectomy in large vessel occlusion strokes leads to better outcomes than intravenous thrombolysis alone: a 'real world' applicability of the recent trials. Interv Neurol. (2016) 5:101–10. 10.1159/000445809 27781037 PMC5075836

[7] Kara B Selcuk HH Erbahceci Salik A Zalov H Yildiz O Gul G. Single-center experience with the tigertriever device for the recanalization of large vessel occlusions in acute ischemic stroke. J Neurointerv Surg. (2019) 11:455–9. 10.1136/neurintsurg-2018-014196 30262656

[8] Procházka V Jonszta T Czerny D Krajca J Roubec M Hurtikova E. Comparison of mechanical thrombectomy with contact aspiration, stent retriever, and combined procedures in patients with large-Vessel occlusion in acute ischemic stroke. Med Sci Monit. (2018) 24:9342–53. 10.12659/MSM.913458 30578729 PMC6320656

[9] Ho FL Chapot R. Removal of distal fragments of liquid embolic agents during arteriovenous malformation embolization using the TIGERTRIEVER 13: a technical report. J Neurointerv Surg. (2020) 12:794–7. 10.1136/neurintsurg-2019-015474 32024783

[10] Powers WJ Rabinstein AA Ackerson T Adeoye OM Bambakidis NC Becker K. Guidelines for the early management of patients with acute ischemic stroke: 2019 Update to the 2018 Guidelines for the Early Management of Acute Ischemic Stroke: a Guideline for Healthcare Professionals From the American Heart Association/American Stroke Association. Stroke. (2019) 50:e344-e418. 10.1161/STR.0000000000000211 31662037

[11] Turc G Bhogal P Fischer U Khatri P Lobotesis K Mazighi M. European stroke organisation (ESO)- european society for minimally invasive neurological therapy (ESMINT) guidelines on mechanical thrombectomy in acute ischemic stroke. J Neurointerv Surg. (2019) 11:535–8. 10.1136/neurintsurg-2018-014568 31152058

[12] Turc G Bhogal P Fischer U Khatri P Lobotesis K Mazighi M. European stroke organisation (ESO) - european society for minimally invasive neurological therapy (ESMINT) guidelines on mechanical thrombectomy in acute ischaemic strokeEndorsed by stroke alliance for europe (SAFE). Eur Stroke J. (2019) 4:6–12. 10.1177/2396987319832140 31165090 PMC6533858

[13] Zaidat OO Yoo AJ Khatri P Tomsick TA von Kummer R Saver JL. Recommendations on angiographic revascularization grading standards for acute ischemic stroke: a consensus statement. Stroke. (2013) 44:2650–63. 10.1161/STROKEAHA.113.001972 23920012 PMC4160883

[14] Fiorelli M Bastianello S von Kummer R del Zoppo GJ Larrue V Lesaffre E. Hemorrhagic transformation within 36 hours of a cerebral infarct: relationships with early clinical deterioration and 3-month outcome in the european cooperative acute stroke study i (ECASS I) cohort. Stroke. (1999) 30:2280–4. 10.1161/01.STR.30.11.2280 10548658

[15] Turk AS Spiotta A Frei D Mocco J Baxter B Fiorella D. Initial clinical experience with the ADAPT technique: a direct aspiration first pass technique for stroke thrombectomy. J Neurointerv Surg. (2014) 6:231–7. 10.1136/neurintsurg-2013-010713 23624315

[16] Saver JL Jahan R Levy EI Jovin TG Baxter B Nogueira RG. Solitaire flow restoration device versus the merci retriever in patients with acute ischaemic stroke (SWIFT): a randomised, parallel-group, non-inferiority trial. Lancet. (2012) 380:1241–9. 10.1016/S0140-6736(12)61384-1 22932715

[17] Grech R Pullicino R Thornton J Downer J. An efficacy and safety comparison between different stentriever designs in acute ischaemic stroke: a systematic review and meta-analysis. Clin Radiol. (2016) 71:48–57. 10.1016/j.crad.2015.09.011 26597570

[18] Nogueira RG Lutsep HL Gupta R Jovin TG Albers GW Walker GA. Trevo versus merci retrievers for thrombectomy revascularisation of large vessel occlusions in acute ischaemic stroke (TREVO 2): a randomised trial. Lancet. (2012) 380:1231–40. 10.1016/S0140-6736(12)61299-9 22932714 PMC4176618

[19] Ducroux C Piotin M Gory B Labreuche J Blanc R Ben Maacha M. First pass effect with contact aspiration and stent retrievers in the aspiration versus stent retriever (ASTER) trial. J Neurointerv Surg. (2020) 12:386–91. 10.1136/neurintsurg-2019-015215 31471527 PMC7146919

[20] Frei D Gerber J Turk A McPherson M Heck D Hui F. The SPEED study: initial clinical evaluation of the penumbra novel 054 reperfusion catheter. J Neurointerv Surg. (2013) 5 Suppl 1:i74–6. 10.1136/neurintsurg-2012-010585 23299104

[21] Kowoll A Weber A Mpotsaris A Behme D Weber W Direct aspiration first pass technique for the treatment of acute ischemic stroke: initial experience at a European stroke center. J Neurointerv Surg. (2016) 8:230–4. 10.1136/neurintsurg-2014-011520 25583533

[22] Primiani CT Vicente AC Brannick MT Turk AS Mocco J Levy EI. Direct aspiration versus stent retriever thrombectomy for acute stroke: a systematic review and meta-analysis in 9127 patients. J Stroke Cerebrovasc Dis. (2019) 28:1329–37. 10.1016/j.jstrokecerebrovasdis.2019.01.034 30772159

[23] Qin C Shang K Xu SB Wang W Zhang Q Tian DS. Efficacy and safety of direct aspiration versus stent-retriever for recanalization in acute cerebral infarction: a PRISMA-compliant systematic review and meta-analysis. Medicine (Baltimore). (2018) 97:e12770. 10.1097/MD.0000000000012770 30313091 PMC6203566

